# Supplementary material for: Soil classification predicts differences in prokaryotic communities across a range of geographically distant soils once pH is accounted for
Source: Sci Rep. 2017 Mar 28;7:45369. doi: 10.1038/srep45369 (PMC5368985; doi:10.1038/srep45369)
Supplement: Supplementary Information [file srep45369-s1.pdf]

Supplementary Information

Soil classification predicts differences in prokaryotic communities across a range of geographically distant soils once pH is accounted for

Authors: Rachel Kaminsky, Blandine Trouche, and Sergio E. Morales
Department of Microbiology and Immunology, Otago School of Medical Sciences, University of Otago, Dunedin, New Zealand

Supplementary Figures

Figure S1: Relationship between pH and diversity metrics.
Figure S2: ANOSIM test for correlations between land use and Bray-Curtis distances.
Figure S3: Hierarchical clustering for all sample data.
Figure S4: ANOSIM test for correlations between soil classification and Bray-Curtis distances.
Figure S5: Hierarchical clustering for high country sample data.
Figure S6: Hierarchical clustering for sheep and beef sample data.
Figure S7: Hierarchical clustering for dairy sample data.
Figure S8: Ordinations of regional subgroups (Land use).
Figure S9: Ordinations of regional subgroups (Soil classification).
Figure S10: ANOSIM test for correlations between soil classification and land use and Bray-Curtis distances for South Canterbury soils.
Figure S11: ANOSIM test for correlations between soil classification and land use and Bray-Curtis distances for Southland soils.
Figure S12: ANOSIM test for correlations between soil classification and land use and Bray-Curtis distances for North Canterbury soils.
Figure S13: ANOSIM test for correlations between soil classification and land use and Bray-Curtis distances for Otago soils.

Supplementary Tables

Table S1: Soil physicochemical properties measured in this study. Separate file.
Table S2: Mantel correlations for regions.

Table S3: Taxa that are significantly correlated to different physicochemical parameters presented with statistical support. Separate file.

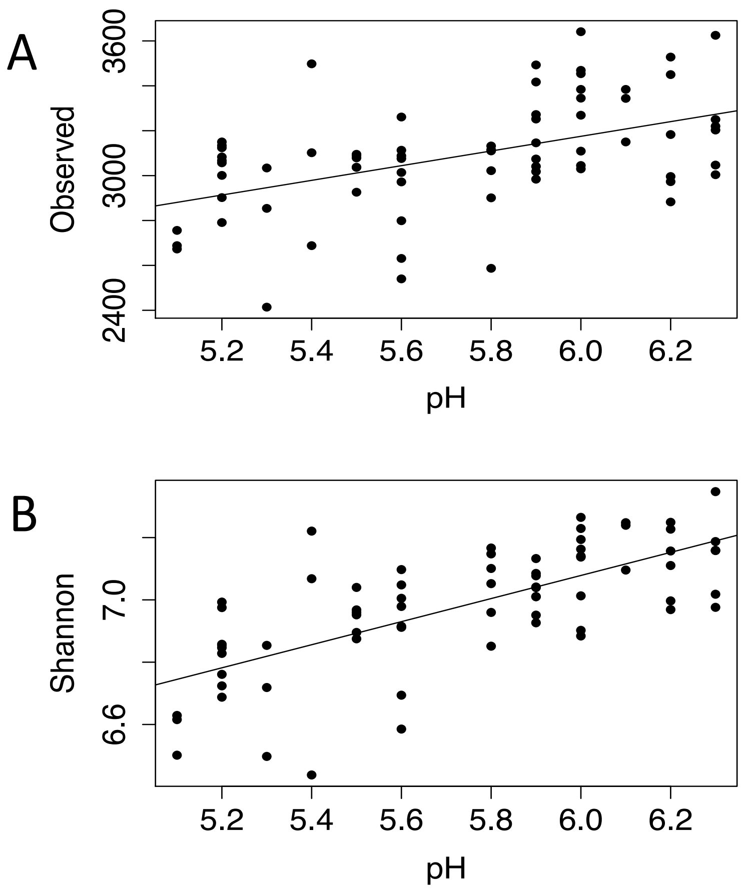

Figure S1: Relationship between pH and diversity metrics. (A) Observed species and (B) Shannon diversity regressed with pH values. Line represents the best-fit linear model.

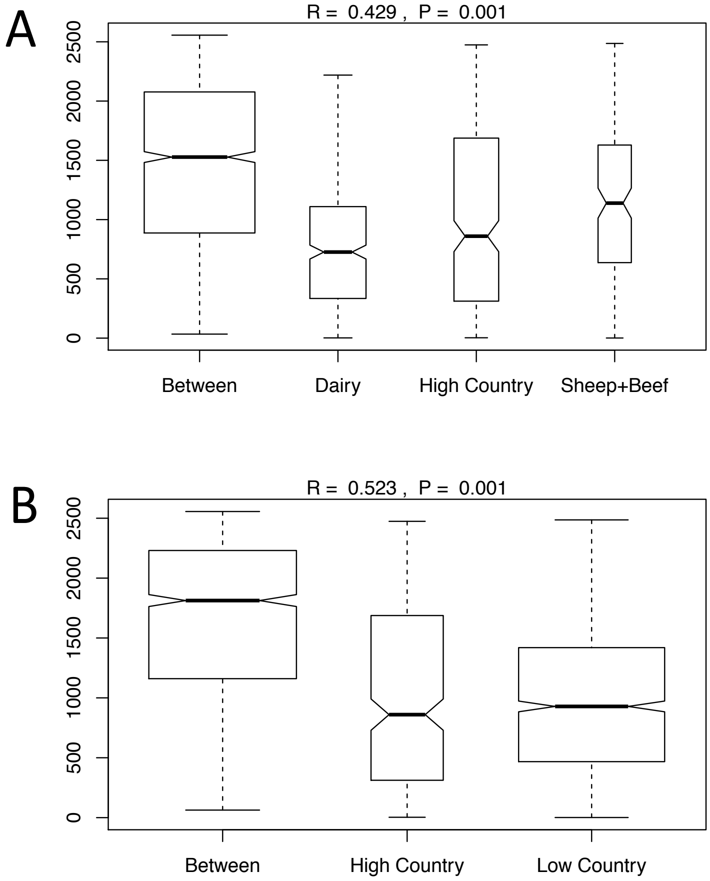

Figure S2: ANOSIM test for correlations between land use and Bray-Curtis distances. (A) Ranked dissimilarities plotted against the three land uses and (B) high country versus low country, where low country includes both sheep and beef and dairy samples. Boxplots are drawn with widths proportional to the square roots of the number of observations in each land use and with 95% confidence intervals.

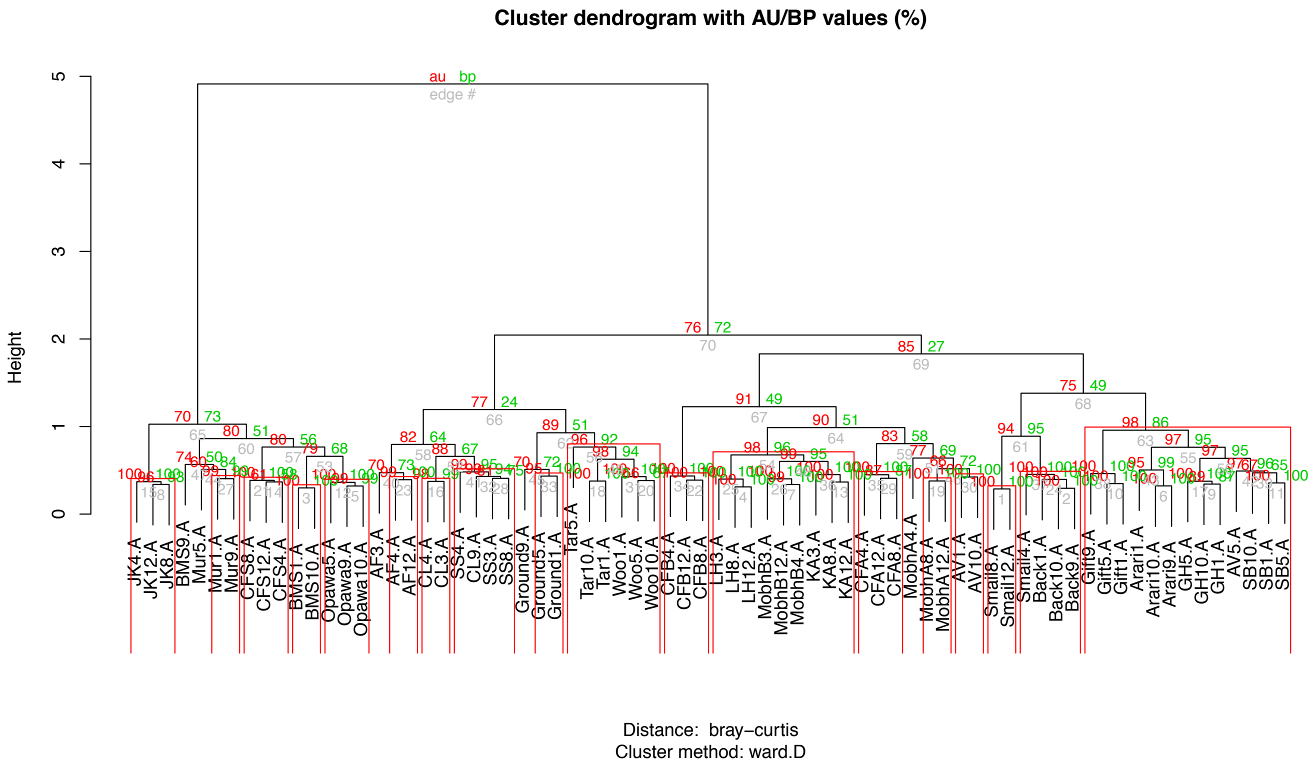

Figure S3: Hierarchical clustering for all sample data. Bray-Curtis distances were used to compare diversity between samples. This revealed two large, weakly supported clusters, with many well-supported sub-clusters. Branches are labeled with an approximately unbiased p-value (red), a bootstrap probability value (green). Bootstrapping was performed at nboot = 1000.

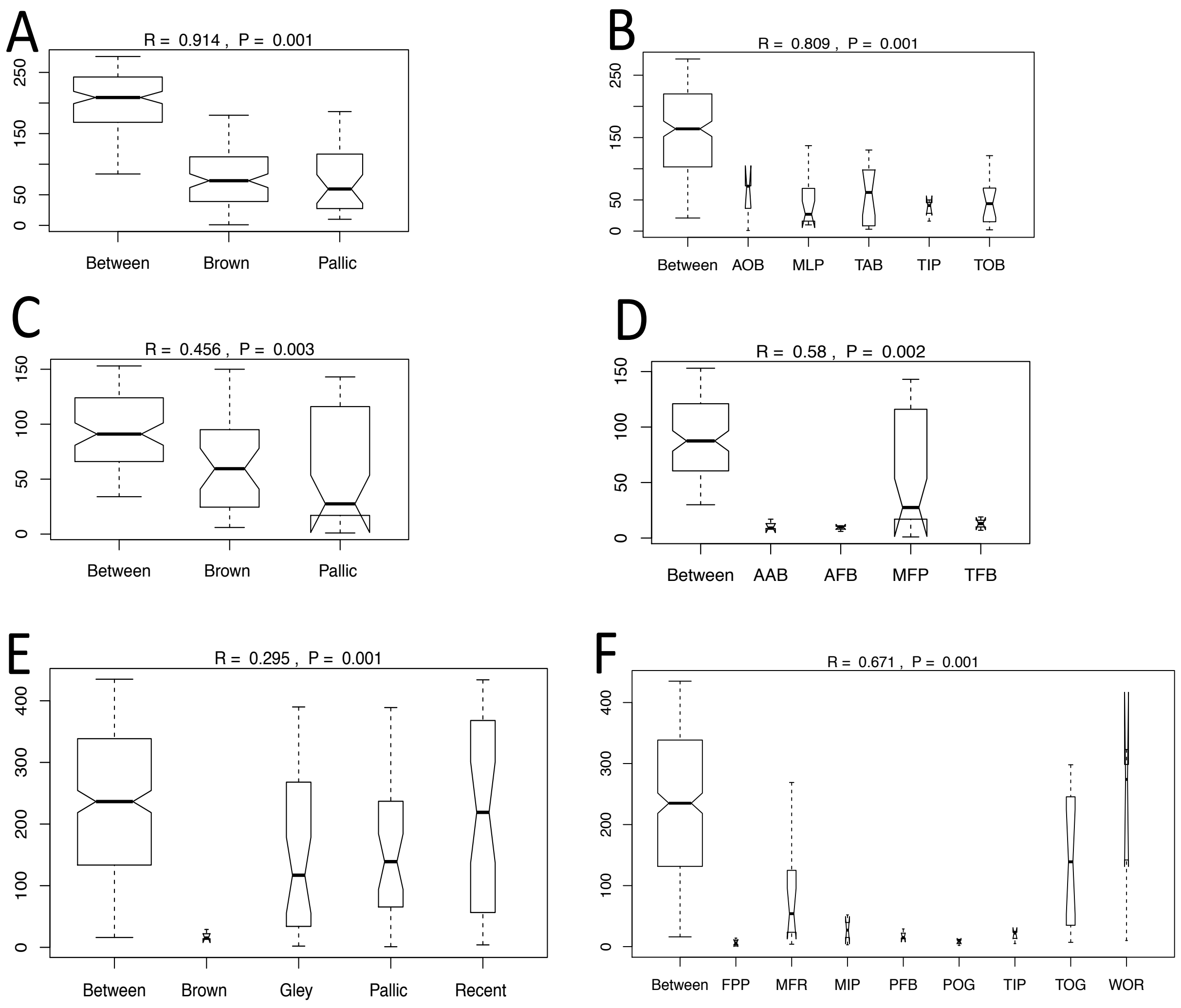

Figure S4: ANOSIM test for correlations between soil classification and Bray-Curtis distances. Ranked dissimilarities plotted against soil order (A, C, E) and soil subgroup (B, D, F) for high country soils (A, B) sheep and beef soils (C, D) and dairy soils (E, F). Boxplots are drawn with widths proportional to the square roots of the number of observations in each land use and with 95% confidence intervals. Soil subgroup abbreviations: AOB = acidic orthic brown; MLP = mottled laminar pallic; TAB = typic acid brown; TIP = typic immature pallic; TOB = typic orthic brown; AAB = acidic-pedal allophanic brown; AFB = acidic firm brown; MFP = mottled fragic pallic; TFB = typic firm brown; FPP = fragic perch-gley pallic; MFR = mottled-acidic fluvial recent; PFB = pallic firm brown; POG = peaty orthic gley; TIP = typic immature pallic; TOG = typic orthic gley; WOR = weathered orthic recent.

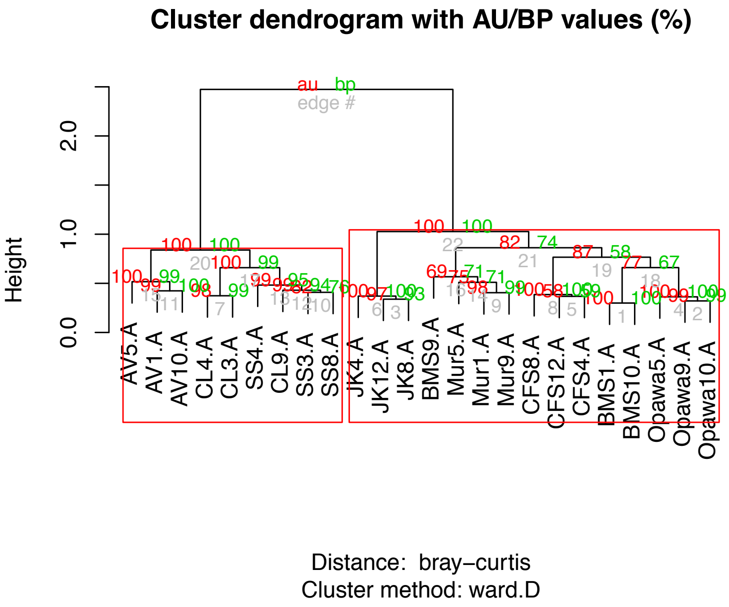

Figure S5: Hierarchical clustering for high country sample data. Bray-Curtis distances were used to compare diversity between samples, revealing two large clusters. Branches are labeled with an approximately unbiased p-value (red), a bootstrap probability value (green). Bootstrapping was performed at nboot = 1000.

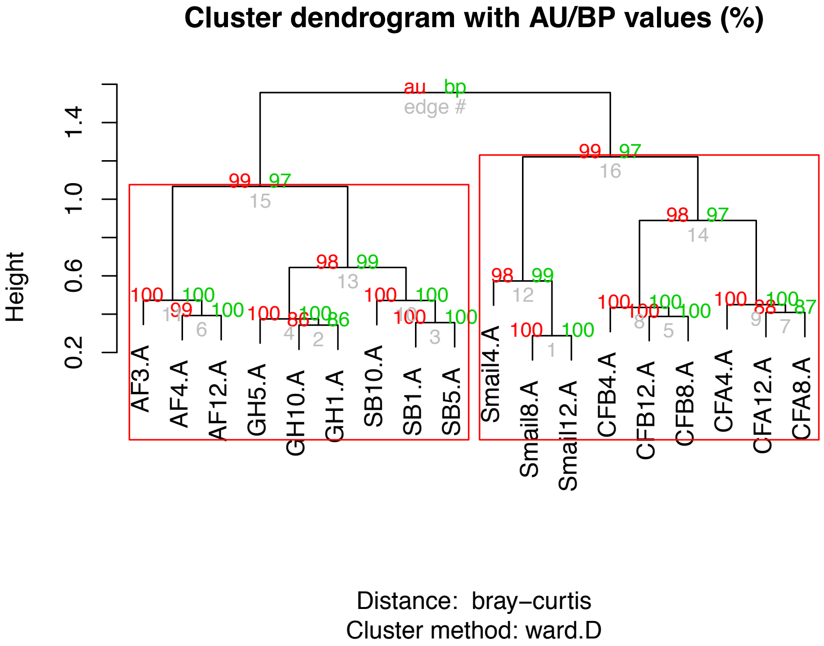

Figure S6: Hierarchical clustering for sheep and beef sample data. Bray-Curtis distances were used to compare diversity between samples, revealing five clusters. Branches are labeled with an approximately unbiased p-value (red), a bootstrap probability value (green). Bootstrapping was performed at nboot = 1000.

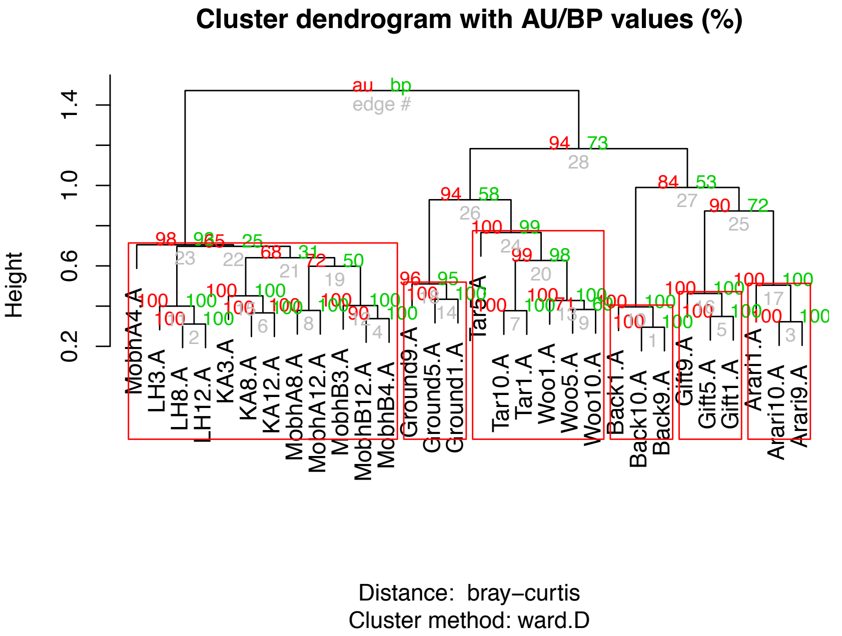

Figure S7: Hierarchical clustering for dairy sample data. Bray-Curtis distances were used to compare diversity between samples, revealing two large clusters. Branches are labeled with an approximately unbiased p-value (red), a bootstrap probability value (green). Bootstrapping was performed at nboot = 1000.

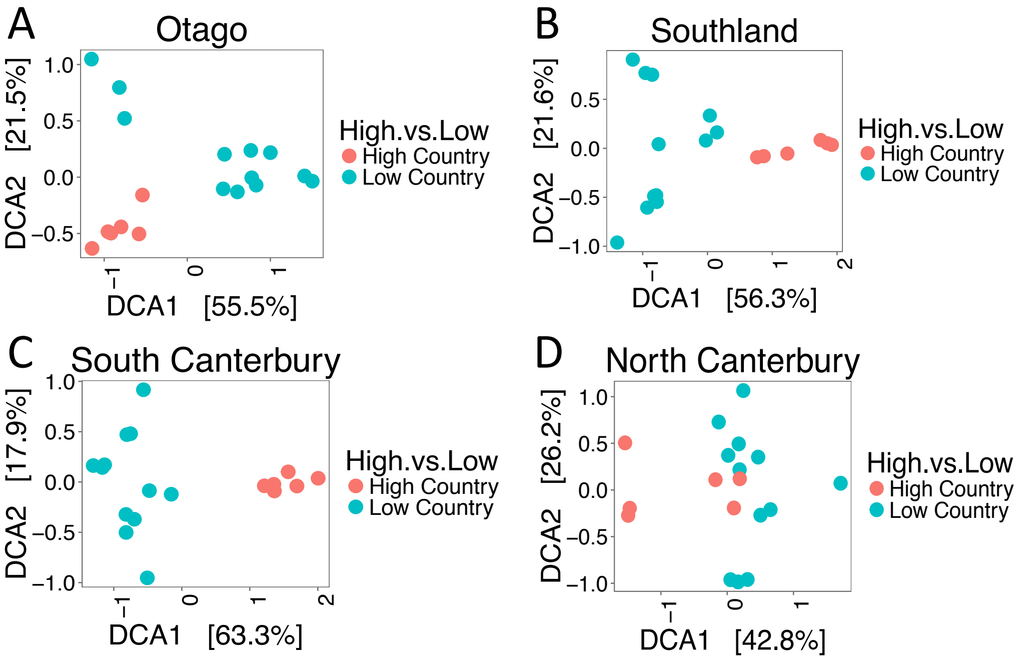

Figure S8: Ordinations of regional subgroups. DCA plots on a Bray-Curtis distance matrix showing the relationship between land use (simplified to low country versus high country) and and Otago soils (A), Southland soils (B), South Canterbury soils (C) and North Canterbury soils (D). Land use is indicated by color.

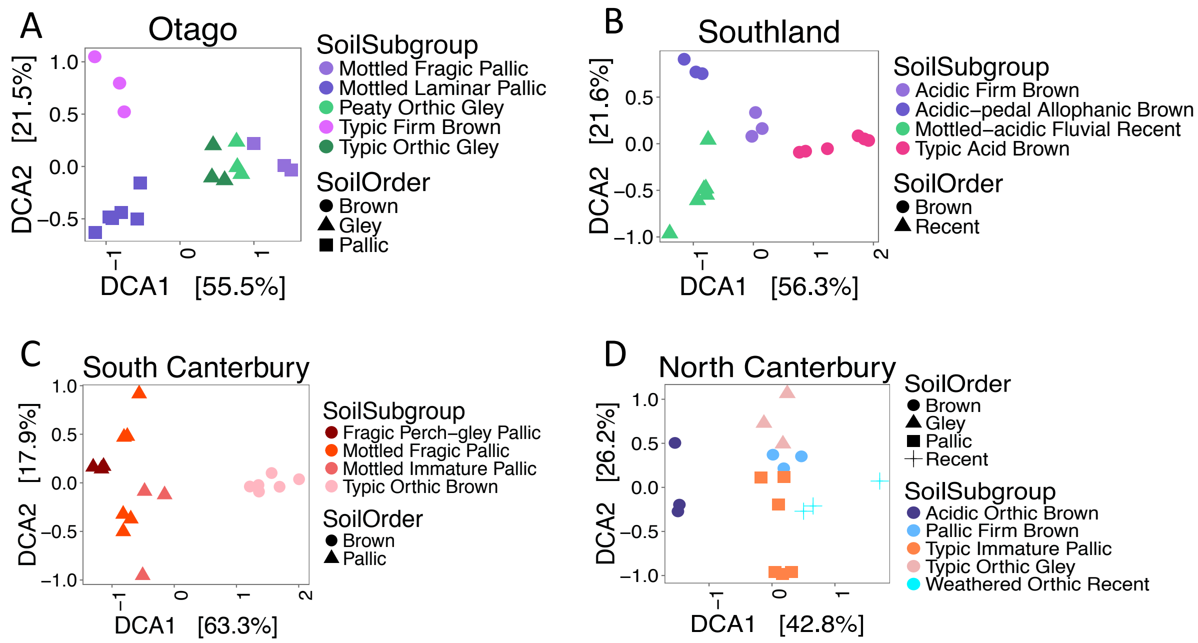

Figure S9: Ordinations of regional subgroups. DCA ordination based on Bray-Curtis showing relationships between soil classification and Otago soils (A), Southland soils (B), South Canterbury soils (C) and North Canterbury soils (D). Soil subgroup is indicated by color and soil order is indicated by shape.

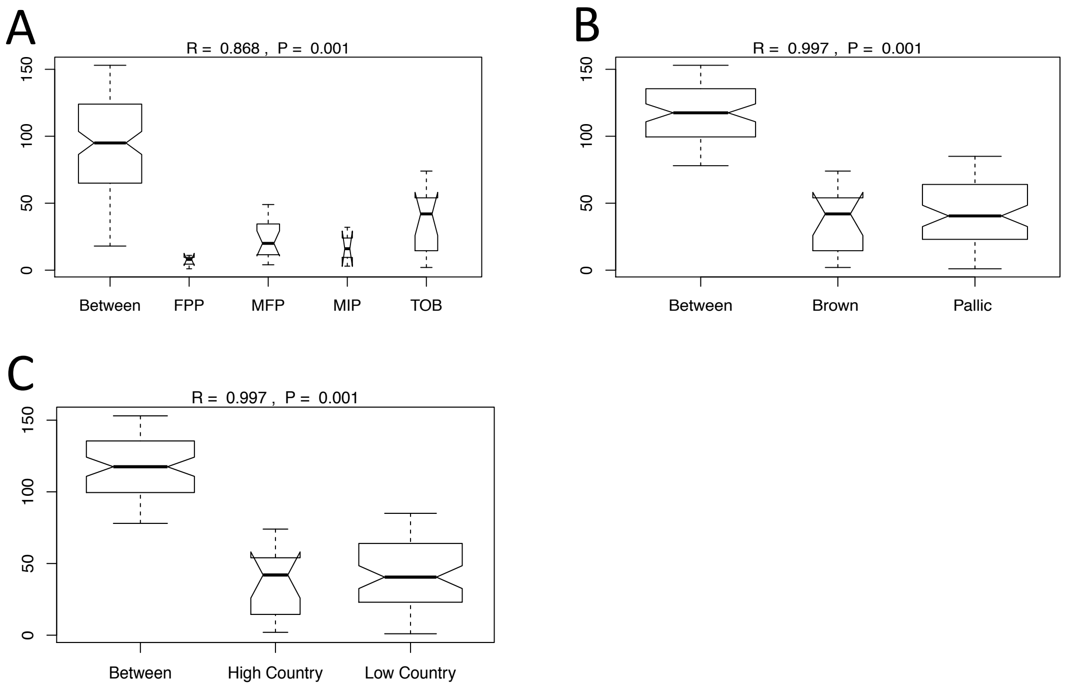

Figure S10: ANOSIM test for correlations between soil classification and land use and Bray-Curtis distances for South Canterbury soils. (A) Ranked dissimilarities plotted against soil subgroup, (B) soil order and (C) land use. Boxplots are drawn with widths proportional to the square roots of the number of observations in each land use and with 95% confidence intervals.

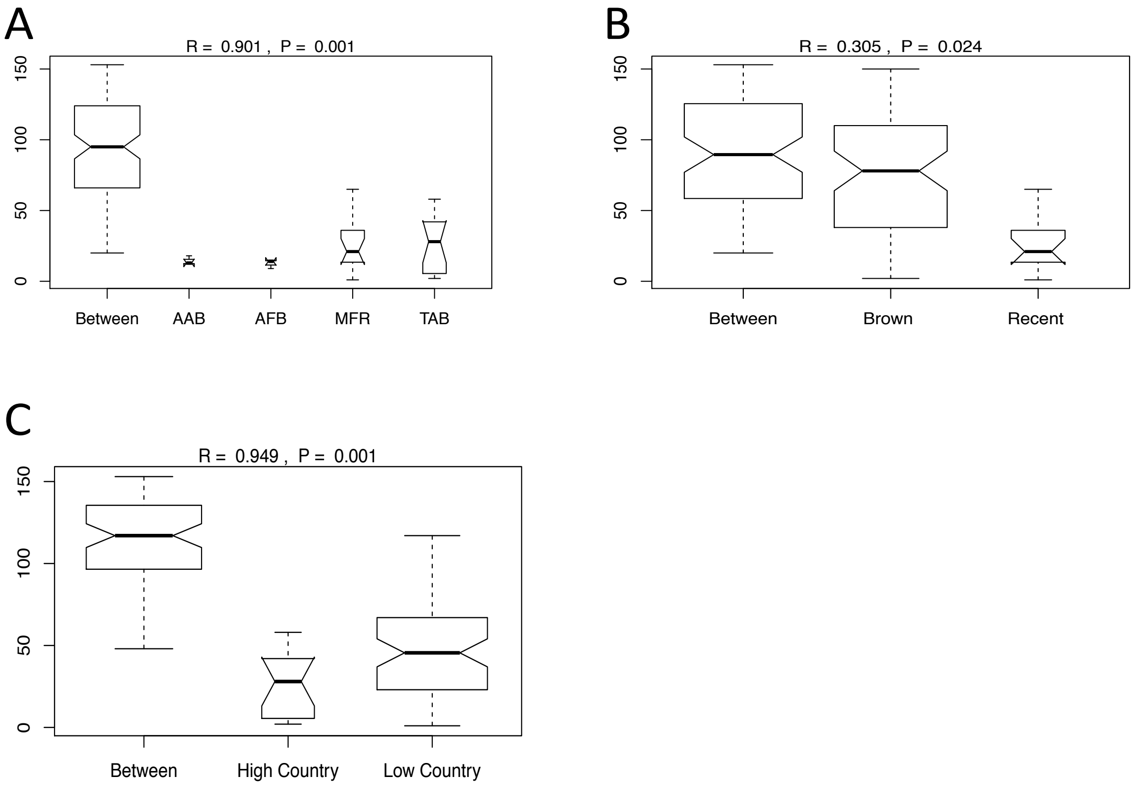

Figure S11: ANOSIM test for correlations between soil classification and land use and Bray-Curtis distances for Southland soils. (A) Ranked dissimilarities plotted against soil subgroup, (B) soil order and (C) land use. Boxplots are drawn with widths proportional to the square roots of the number of observations in each land use and with 95% confidence intervals. Soil subgroup abbreviations: TAB = typic acid brown; AAB = acidic-pedal allophanic brown; AFB = acidic firm brown; MFR = mottled-acidic fluvial recent

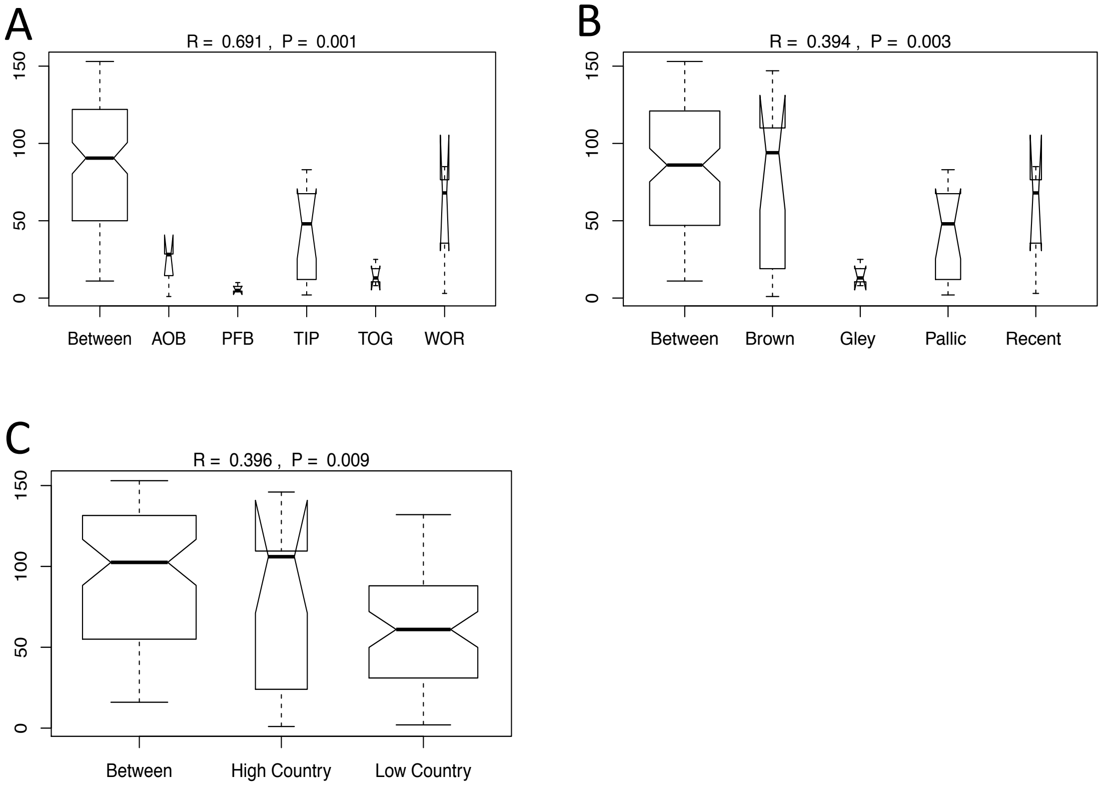

Figure S12: ANOSIM test for correlations between soil taxonomy and land use and Bray-Curtis distances for North Canterbury soils. (A) Ranked dissimilarities plotted against soil subgroup, (B) soil order and (C) land use. Boxplots are drawn with widths proportional to the square roots of the number of observations in each land use and with 95% confidence intervals. Soil subgroup abbreviations: AOB = acidic orthic brown; PFB = pallic firm brown; TIP = typic immature pallic; TOG = typic orthic gley; WOR = weathered orthic recent

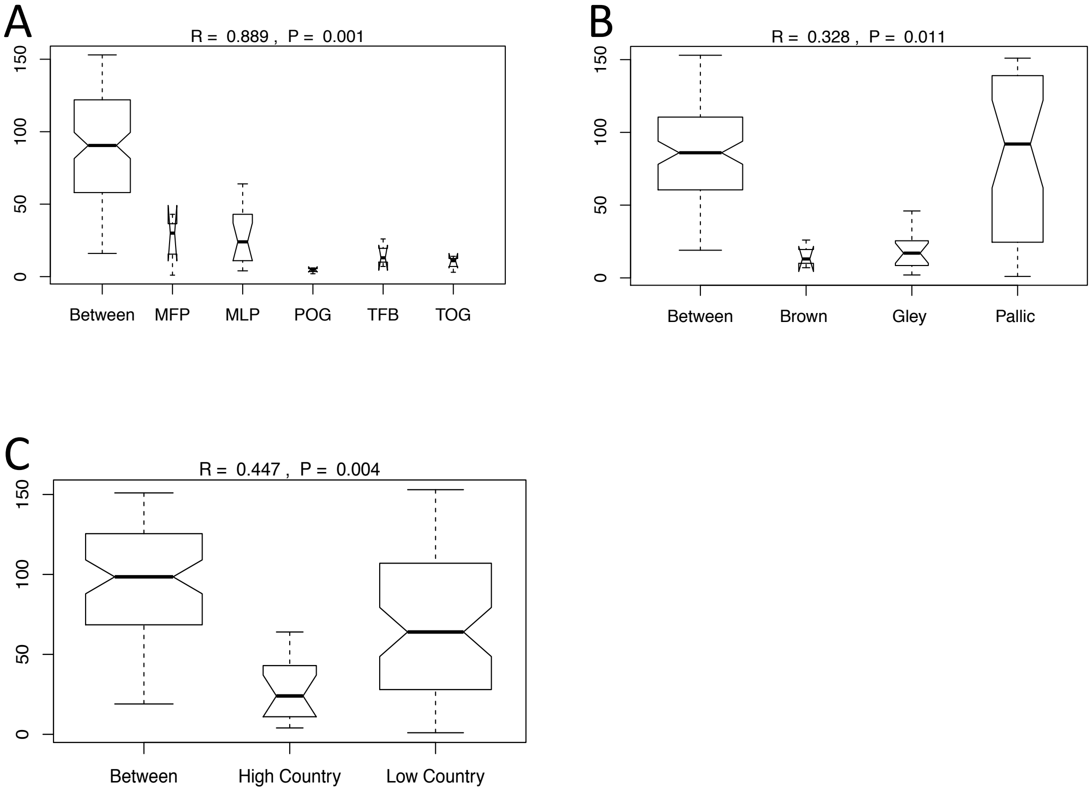

Figure S13: ANOSIM test for correlations between soil taxonomy and land use and Bray-Curtis distances for Otago soils. (A) Ranked dissimilarities plotted against soil subgroup, (B) soil order and (C) land use. Boxplots are drawn with widths proportional to the square roots of the number of observations in each land use and with 95% confidence intervals. Soil subgroup abbreviations: MFP = mottled fragic pallic; MLP = mottled laminar pallic; POG = peaty orthic gley; TFB = typic firm brown; TOG = typic orthic gley

Table S2: Mantel Correlations between pH and Bray-Curtis distances for within-region communities

**Region**	**Mantel R^2**	**p-value**
North Canterbury	0.37	<0.002
South Canterbury	0.85	<0.001
Otago	0.48	<0.001
Southland	0.81	<0.001
